# Granular Data Access Control with a Patient-Centric Policy Update for Healthcare

**DOI:** 10.3390/s21103556

**Published:** 2021-05-20

**Authors:** Fawad Khan, Saad Khan, Shahzaib Tahir, Jawad Ahmad, Hasan Tahir, Syed Aziz Shah

**Affiliations:** 1 Department of Information Security, National University of Sciences and Technology, Sector H-12, Islamabad 44000, Pakistan; shahzaib.tahir@mcs.edu.pk (S.T.); hasan.tahir@seecs.edu.pk (H.T.); 2 Department of Computer Science & IT, Sarhad University of Science and Information Technology, Peshawar 25000, Pakistan; amlms1.de@suit.edu.pk; 3 School of Computing, Edinburgh Napier University, Edinburgh EH11 4BN, UK; J.Ahmad@napier.ac.uk; 4 Faculty Research Centre for Intelligent Healthcare, Coventry University, Coventry CV1 5FB, UK; syed.shah@coventry.ac.uk

**Keywords:** multi message, hierarchal, policy update, constant computations, constant size ciphertext

## Abstract

Healthcare is a multi-actor environment that requires independent actors to have a different view of the same data, hence leading to different access rights. Ciphertext Policy-Attribute-based Encryption (CP-ABE) provides a one-to-many access control mechanism by defining an attribute’s policy over ciphertext. Although, all users satisfying the policy are given access to the same data, this limits its usage in the provision of hierarchical access control and in situations where different users/actors need to have granular access of the data. Moreover, most of the existing CP-ABE schemes either provide static access control or in certain cases the policy update is computationally intensive involving all non-revoked users to actively participate. Aiming to tackle both the challenges, this paper proposes a patient-centric multi message CP-ABE scheme with efficient policy update. Firstly, a general overview of the system architecture implementing the proposed access control mechanism is presented. Thereafter, for enforcing access control a concrete cryptographic construction is proposed and implemented/tested over the physiological data gathered from a healthcare sensor: shimmer sensor. The experiment results reveal that the proposed construction has constant computational cost in both encryption and decryption operations and generates constant size ciphertext for both the original policy and its update parameters. Moreover, the scheme is proven to be selectively secure in the random oracle model under the q-Bilinear Diffie Hellman Exponent (q-BDHE) assumption. Performance analysis of the scheme depicts promising results for practical real-world healthcare applications.

## 1. Introduction

Cloud is a platform that provides on-demand availability, ubiquitous access to the data and a shared pool of configurable computing resources [1]. The outsourcing of data on the cloud is governed primarily through the principles of data sharing and data management. The benefits that the cloud offers prompt various organizations to outsource their data with reduced security management costs. The organizations and sectors that have already embrace the cloud and further explore its use cases include healthcare, telecommunication, and real-estate. Although the cloud offers many benefits, the remote third-party cloud servers are an active target as hackers may succeed to bypass their security firewalls to get unauthorized access. Even the cloud itself may be malicious and may try to exploit potential vulnerabilities to access the data or grant access to unauthorized users.

Healthcare is a collaborative environment that involves many organizations including pharmaceuticals, hospitals, and insurance companies, and therefore multiple users/multiple actors with different roles are accessing the same resources. Access to a patient’s health record is required for proper diagnosis and prescription [2], insurance claims and data analytics [3]. Since the patients’ health data is sensitive in nature and requires proper management to avoid privacy breaches. To be more precise, the access and interaction of multiple users to the same data gives rise to the possibility of data theft. Therefore, the scope of this research is limited to healthcare in which threats related to unauthorized access are prevented through efficient handling of patients’ data and access rights by limiting unauthorized data access. To attain these goals, a promising solution is the Ciphertext Policy-Attribute-based Encryption (CP-ABE) [4] which has been considered for enforcing cryptographic access control on data. Using CP-ABE the data owner can enforce an access policy in a ciphertext using attributes, and any user conforming to the policy can access the data. ABE either CP-ABE or Key Policy-ABE (KP-ABE) is a one-to-many access control paradigm, that grants the same data access to multiple sets of users upon successful satisfaction of a policy by them.

In existing healthcare access control schemes [5,6,7,8,9], CP-ABE generates the same data for all users due to the fact that against every message a single ciphertext is generated and all the authorized users can have access to that same data. To elaborate, users satisfying individual attribute sets of a monotonic access policy in CP-ABE have access to the same data. Consider a scenario where doctors from a hospital have access to all the patient’s sensitive healthcare data, while the secondary stakeholders consisting of nurses and pharmaceutical firms are restricted to a limited chunk of insensitive data. This granular data access cannot be attained with the conventional CP-ABE mechanisms. Instead, a multi message CP-ABE is required in this context, so that multiple messages, i.e., hierarchically driven keys are encrypted with it for the same data access. More details regarding hierarchical keys derivation is presented Section 6.1. Another limitation of existing healthcare schemes [5,6,7,8,9] is their nature as they are based on single attribute authority, however, in real life attributes can be from multiple stakeholders belonging to different authority domains like hospitals, and universities.

We remark that in the existing health-centric schemes [5,6,7,8,9], the policies are static and predefined. Due to the absence of a policy update feature, CP-ABE cannot be considered as a complete access control enforcement tool. This issue motivates to dynamically increase or decrease the privileges of specific users over certain sets of files. However, if the process of policy update somehow tries to reduce or cancel the access rights of some users over particular attributes, they might refuse an update in their attribute keys to decrypt data even after the policy update. Hence, to address the problem of non-cooperation from users; existing approaches focus to update keys for all unrevoked users in order to update the policy [10]. However, this solution reduces efficiency because the number of revoked attributes is generally a few; therefore, most of the effort is concentrated on unrevoked attributes for a policy update. Moreover, it should not always be the case that attributes are just only revoked from a policy; instead, mechanisms should be developed to cater for both the addition and the revocation of attributes in policy updates. Another issue to be addressed should be that the underlying CP-ABE should have constant computation costs to accommodate the resource-constrained devices. To resolve the above-mentioned issues, our contributions are enlisted below.

### 1.1. Our Contributions

To simultaneously address the inclusion of hierarchical access control to CP-ABE and to dynamically update the access policy privileges with constant computation costs, this paper makes the following contributions:

- This research gives the notion of Patient-Centric Multi Message Ciphertext Policy-Attribute Based Encryption with Efficient Policy Update (PC-MM-CP-ABE-EPU).
- The proposed construction addresses the inclusion of hierarchical access control to CP-ABE and dynamically updates the access policy with constant computational overhead.
- A comprehensive security and performance analysis of the proposed construction is presented to depict its effectiveness for dynamic access control in healthcare.
- For security, we prove the proposed PC-MM-CP-ABE-EPU scheme to be selectively secure in the random oracle model under the q-Bilinear Diffie Hellman Exponent (q-BDHE) assumption.
- This paper also studies the feasibility of the proposed scheme in the healthcare sector, where a patient can utilize it to specify access rights to his confidential data for doctors, nurses, and insurance companies.
- For performance, real data is generated through a body wearable physiological sensor called the Shimmer [11]. The sensor is embedded with Micro-Electrical Mechanical System (MEMS) and physiological sensing components. The proposed scheme is tested using the data collected via the sensor.

### 1.2. Paper Organization

Rest of the paper is organized as follows. Section 2 highlights the existing works relating to the evolvement of attribute based encryption in general, and particularly for enforcing access control of data in healthcare. System Architecture is presented in Section 3, which discusses the roles of various actors in healthcare along with data access control policy specification. The preliminary cryptographic definitions, hardness assumptions, and concepts used to define the proposed PC-MM-CP-ABE-EPU scheme is stated in Section 4. The syntax and security model of the proposed PC-MM-CP-ABE-EPU scheme is listed in Section 5. Section 6, details the proposed cryptographic scheme for patient centric access control provision. In Section 7, we present the performance and security analysis of the scheme. Section 8 concludes the paper.

## 2. Related Work

After the notion of ABE was formalized by Sahai and Water [12], Bethencourt et al. [4] proposed its variant named as CP-ABE. The user decryption keys corresponded to attributes, while ciphertext was related to a policy defined over attributes in CP-ABE. However, the scheme [4] was proven secure in the generic group model. Later, Cheung and Newport [13] proposed a CP-ABE scheme to be secure in the standard model based on AND based access structure. The first decentralized multi authority CP-ABE [14] construction was formalized by Lewko and Water. The notion of multi message CP-ABE for providing access control to scalable media is proposed in [15] by extending the work of [4]. Later, Khan et al. [16] proposed multi message CP-ABE with multiple authorities working in a decentralized manner. However, both the schemes are proven secure in generic group and random oracle model. Zhang et al. [17,18] addressed the user’s attributes information leakage issues.

In [19], each attribute has a feature expiry time. Attribute authority updates keys periodically according to this time parameter. Bethencourt et al. [4] comments that the expiry time should vary from one user to another, and be independent of the user’s attributes. The authors in [20] introduced the concept of the users list so that even if any particular user satisfies the policy, but is excluded from the authorized list, he cannot have access to data. Further, the idea of the user ID revocation list is presented in [21]. However, both the time and ID based access control methodologies suffer from potential problems i.e., for the system controlling user privilege rights with respect to time needs to define the expiry time during the generation of user attribute decryption keys. Similarly, the authorized ID list needs to be generated along with ciphertext in encryption operation. Hence, any dynamic change of access control cannot be provisioned by employing these concepts. Yang et al. [10,22] proposed the concept of dynamic change of privileges by updating both the ciphertext and user decryption keys in case of attribute revocation. However, the incurred cost for both updating the ciphertext and user keys for non-revoked users is too much for practical considerations. Moreover, as stated in [23], the scheme presented in [22] is not collusion resistant after an update for attribute revocation is performed. Some other recent works tackling the issue of policy updating, i.e., addition and revocation of attributes are [24,25,26,27,28]. The Linear Secret Sharing Scheme (LSSS) matrix based access structure is employed for ciphertext generation and update policy in [24,25]. Moreover, the scheme in [24] is based on composite order groups and proved to be adaptively secure in standard model. Jiang et al. [26] presented the notions of two constructions separately for both attribute addition and attribute revocation selectively secure under the MSE-DDH assumption. In [27], the authors proposed a threshold policy update based CP-ABE. Signcryption based CP-ABE with policy update and outsourced computations is proposed in [28]. However, the computational costs of these schemes [26,27,28] is much and not suitable for resource-constrained devices. The authors [29] have effectively demonstrated the significance of ABE for resource-constrained IoT devices. Some other enhanced CP-ABE schemes [30] with variant features include attribute based proxy re-encryption [7,31], accountable CP-ABE [32], online/offline CP-ABE [5,7] and outsourced CP-ABE [33].

ABE has been employed in healthcare domain to address concerns relating to resource constrained client [5], doctor centric access control [6] and searchable trapdoor for hospital data [8,9] as seen from Table 1. However, existing schemes failed to grant patients with user-centric access control and policy update features as seen from Table 1. Moreover, all existing schemes are based on a single attribute authority, making it less scalable for autonomous organizations. Also, all existing schemes encrypt only a single message over a policy, thereby limiting the provision of hierarchical access control. All these issues are addressed in our proposed scheme for which we have designed a system architecture along with the cryptographic scheme as discussed in Section 3 and Section 6 that can be easily employed in any healthcare facility requiring patient-centric access control. Another similar line of work for health-centric access control provision is Georgakakis et al. [34]. The authors in [34] proposed a generic location and time aware role-based access control mechanism for healthcare. Moreover, in emergency cases, the data access can be provisioned by the proposed “break the glass” notion to allow users to access data that they were not entitled to access under normal conditions. However, the mechanism is generic in nature, and no concrete cryptographic scheme is detailed to enforce access control.

## 3. System Architecture

This section is about the application of the proposed system in healthcare context with the help of a use-case scenario. This use-case presents a model according to which access rights and data security can be managed in a healthcare vicinity. To accomplish the desired objectives, this model is divided into four phases namely, data collection, policy specification/update and data aggregation, outsourced data access control and policy update, and data access whose details are provided below. Following that, the actors like a patient, doctor, professor, and insurance agent, who will use the system for secure access of data are discussed. Thereafter, core functions of the model including contextual policy specification and its update are explained. Finally, the functionality of other major components of the model involved in the smooth delivery of health facilities including attributes authorities (AA), cloud, and gateway are detailed. Finally, the security requirements that will be achieved by the proposed system are discussed at the end of this section.

**Figure 1:**
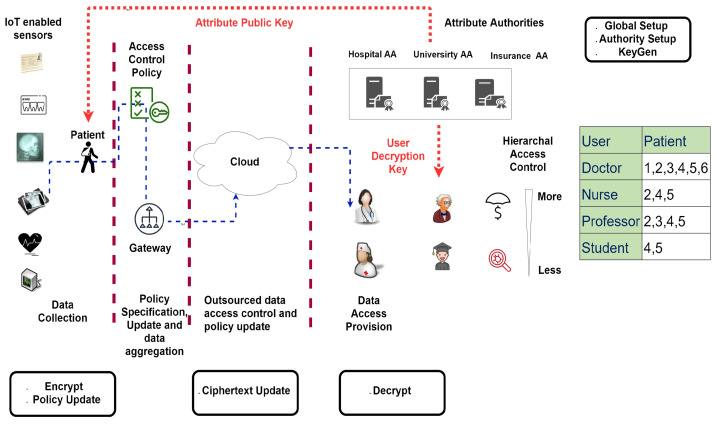
System Model of PC-MM-CP-ABE-EPU.

In a healthcare scenario, the role of IOT-enabled sensors is pivotal as they help in collecting, retrieving, analyzing, and monitoring patients’ medical data in real-time, which eventually helps in dealing with chronic diseases. The proposed system is a novel patient-centric multi-layered model to secure access of data present in semi-trusted servers. The proposed model is a suite of mechanisms, which provides hierarchical access control by considering access control policy defined by patient based on the actor’s attributes. It leverages CP-ABE techniques to secure personal health data of patients being outsourced to the cloud and other related servers. Figure 1 depicts the proposed model of PC-MM-CP-ABE-EPU which works in four phases including data collection, policy specification update and data aggregation, outsourced data access control and policy update, and data access provision. The first phase is concerned with data collection from different IoT-enabled sensory devices. The next phase aggregates data at the gateway node. Moreover, access policy along with policy updates have also been specified in this phase. The third phase outsources data being controlled by the access control policy modules to the cloud. The last phase is all about accessing patients’ data by doctors, nurses, professors, students and insurance companies. A brief description of the involved actors implementing the above-mentioned functionalities is as follows:

- **Patient:** An entity seeking some medical treatment. This entity is responsible for encrypting data and defining/updating policy. For this, the patient executes Encrypt and Policy Update algorithms of PC-MM-CP-ABE-EPU, which are detailed in Section 6.2, and can be from Figure 1.
- **Doctor:** A medical practitioner providing general treatment.
- **Nurse:** A clinical personnel providing treatment and care to patients.
- **Professor:** An individual accessing medical data for research and development.
- **Student:** An entity requesting access to a subset of patient data for research.
- **Insurance company:** It assists patients in covering health expenses.
- **Insurance agent:** It assists patients by offering different healthcare plans based on their health condition and income.

In order to access the patient’s data, all other actors except patient execute the Decrypt algorithm of PC-MM-CP-ABE-EPU, which are detailed in Section 6.2, and can be from Figure 1. Actors within a system require special access to the resources. For instance, doctors and nurses attending a particular patient may require access to the IOT-enabled sensors attached to it. Such access is generated based on the attributes of the actor. A cardiologist may entail access to heart monitors or ECG sensors only, while a neurologist acquires Electromyography (EMG), accelerometers, and gyroscopes. Hence, in the case of the proposed model, access would be granted based on the attributes of doctors and nurses. Since the proposed model provides hierarchical access control, therefore, the entities lower in privileges or hierarchy would have fewer rights in comparison to its parent. For instance, a nurse can access limited resources. Considering a scenario where a patient shares his specific data among the various actors including doctor, nurse and professor with the policy defined as (Hospital ∧ Doctor) OR (Hospital ∧ Nurse) OR (University ∧ Professor). Later, the patient decides to allow the student at the university to have access to data for research purposes, and to an insurance agent for claims regarding his medical expenditure. The updated version of the policy is (Hospital ∧ Doctor) OR (Hospital ∧ Nurse) OR (University and Professor) OR (University ∧ Student) OR (Insurance-company ∧ Insurance-agent). Consider a scenario where doctors from a hospital have access to all the patient’s sensitive healthcare data, while the secondary stakeholders consisting of pharmaceutical firms, insurance companies and government are restricted to a limited chunk of insensitive data. This model is based on the concept of hierarchical access control, which means that each entity will acquire data based on its hierarchical position in the network. As shown in Figure 1 doctor can access all sensors of patient whereas nurse can only access 2, 4, and 5. It is because the nurse is lower in the hierarchy in terms of access privileges, and the patient has limited his access rights to certain specific sensors data only. Since the doctor is on top of hierarchy so he can easily view information accessible to his subordinates.

Apart from actors, other major components of the model involved in the smooth delivery of health facilities include attributes authorities (AA), cloud, and gateway whose major tasks are discussed below.

- **Attribute Authority:** It is an entity that generates the public key parameters for contextual attributes (like for doctor and nurse), and assign decryption keys to user’s based on their Global Identifiers (GID) and possessed attributes. All the attribute authorities, like Hospital AA, University AA, Insurance Company AA works in a decentralized manner. The algorithms of PC-MM-CP-ABE-EPU including the Global Setup, Authority Setup, and KeyGen are executed at AA as seen from Figure 1, and are detailed in Section 6.2.
- **Gateway:** It acts as a trusted relay node to the cloud server with the help of a backbone network. The devices transmit generated data to the gateway.
- **Cloud server:** The cloud server is a semi-trusted entity possessing great storage capacities and high computing power. It aims at storing a volume of encrypted data collected from several devices. The algorithm of PC-MM-CP-ABE-EPU namely Ciphertext Update is executed by Cloud as seen from Figure 1, and is detailed in Section 6.2.
- **IoT enabled sensors:** These sensors are connected with the human body and collect biomedical data of patients. Some of these sensors include ECG, blood pressure, EEG, blood glucose, or pulse oximetry. Such sensors transmit biomedical data of patients to device, which will eventually be transmitted to the cloud.
- **Device:** A device with the help of its built-in sensors is efficient enough to sense, process, and communicate data being generated. Due to these capabilities, different objects can be inter-connected over the network. These devices produce and dispense data to the gateway through a wireless communication medium. Since such data is sensitive in nature, so to assure its confidentiality, it is essential for constrained devices to encrypt it.

**Figure 2:**
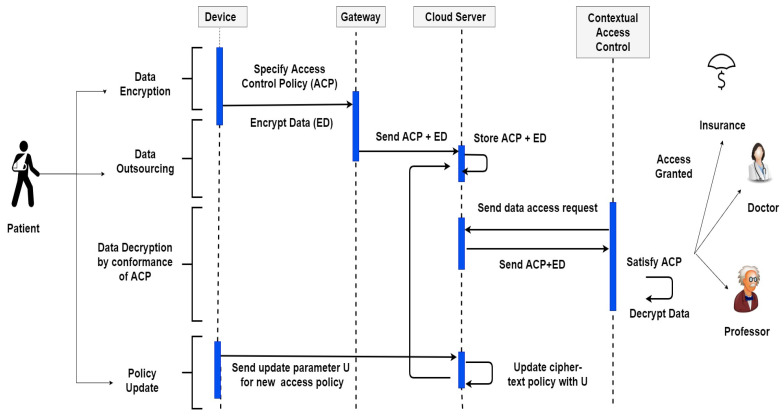
Flow Diagram of Access Control Actions.

The flow of activities of the proposed model is exhibited in Figure 2. However, this model is based on some assumptions. Firstly, the attribute authorities are reliable entities, which can be fully trusted. Secondly, gateway and cloud are honest bodies but are curious. The gateway does not connive with the unauthorized receiver. Cloud will follow the protocol run, but it will try to infer and analyze the encrypted data placed over it. The cloud is considered as an adversary, but we will prove in the security proof, that the challenge ciphertext will be indistinguishable from the perspective of the adversary before and after ciphertext update. The proposed model covers the following aspects of security requirements.

- **Confidentiality and scalability:** As the data produced by the devices contain critical content, therefore it should be kept secured and protected from unauthorized entities and cloud servers. Moreover, the proposed model should be flexible enough to accommodate a large group of authorized users accessing their data.
- **Fine-grained access control:** The senders should define an access control policy for the transmitted data, which can be decrypted by the receivers possessing accurate attribute keys that comply with the access policy in the ciphertext.
- **Collusion resistance:** As the devices and applications accessing the system are not trusted, therefore it is significant to ensure that two or more receivers cannot access data by integrating their attribute keys which they can’t access separately.
- **Secure policy updating:** The algorithm supporting policy update of ciphertext should never disclose critical information to the cloud server.

## 4. Preliminaries

In this section, we detail the definitions of Bilinear Pairing, q-BDHE hardness assumption, and access policy which is used to define a PC-MM-CP-ABE-EPU scheme in the forthcoming sections.

**Definition** **1.** ***Bilinear Pairing** Let G be a multiplicative cyclic group of large prime order $p^{\prime }$, where generator $g\in_{R}G$, and $G_{T}$ is multiplicative cyclic group of same order with its identity denoted by 1. Then, a bilinear pairing e:G x $G\to G_{T}$ is a map with following properties:*

*1.*  *Bilinear: $e(g^{x},g^{y})$ = $e{\left(g,g\right)}^{xy}$$\forall x,y\in Z_{p}$.*
*2.*  *Non-Degenerate: There exists $g_{1},g_{2}\in G$ such that $e(g_{1},g_{2})\neq 1$.*
*3.*  *Computable: Existence of an efficient algorithm to compute $e(g_{1},g_{2})$$\forall g_{1},g_{2}\in G$.*

**Definition** **2.** **q-BDHE** [35] Consider a bilinear group G of prime order p having two independent generators g and h selected at random from it. We represent $y_{g,\alpha ,q}=(g,g_{1},g_{2},\ldots ,g_{q},g_{q+2}$, $,\ldots ,g_{2q})\in G^{2q-1}$ for $g_{i}=g^{\alpha^{i}}$ for an unknown random $\alpha \in Z_{p}^{*}$. An algorithm B which randomly selects β={0,1} has advantage ϵ of solving the q-BDHE problem if $|Pr\left[B\left(g,h,y_{g,\alpha ,q},e\left(g_{q+1},h\right)=1\right)\right]|-|Pr\left[B\left(g,h,y_{g,\alpha ,q},T=1\right)\right]|\geq \epsilon$.

**Definition** **3.** ***Access Policy** A Disjunctive Normal Form (DNF) policy W, namely a ciphertext policy for CP-ABE is a rule that returns either 0 or 1 given a set L of attributes. We say that L satisfies W if and only if W answers 1 on L. We use the notation L⊧W to denote the fact that L satisfies W, and the case of L does not satisfy W is represented by L≠W. Formally, given an access policy $W=[W_{1},W_{2},\ldots ,W_{p^{\prime }}]$ = $\vee_{i\in I_{W}}W_{i}$, where $I_{W}=\left\{i|1\leq i\leq p^{\prime }\right\}$ is a subscript index set of W. Moreover, for 1≤i≤p each of $W_{i}$ = $\left[v_{1},v_{2},\ldots ,v_{m}\right]$ = $\wedge_{j\in I_{X}}v_{j}$, where $I_{X}=\left\{j|1\leq j\leq m\right\}$ is a subscript index set of $W_{i}$. Given a user attribute list $L=[L_{1},L_{2},\ldots ,L_{m}]$, we say that L⊧W if $L_{j}=v_{j}$ for any one attribute set $W_{i}$ of W for $1\leq i\leq p^{\prime }$, and for all 1≤j≤m.*

## 5. Syntax and Security Model

This section details the syntax and security model of our proposed scheme. Notations used throughout the paper are listed in Table 2.

### 5.1. Syntax of PC-MM-CP-ABE-EPU

In this subsection, the algorithms that are part of Patient-Centric multi message CP-ABE with efficient policy update (PC-MM-CP-ABE-EPU) are discussed. Here, we detail only the syntax of the algorithms, the concrete cryptographic construction is presented in Section 6. Referring to Figure 1 in Section 3, the algorithms Global Setup, Authority Setup, KeyGen are executed by attribute authorities, Encrypt and Policy Update is executed by Patient, Ciphertext Update by Cloud, and Decrypt by contextual users like a doctor, nurse, and professor.

**Global Setup**(λ)→GP: Taking the security parameter λ as input, the algorithm outputs the global parameters GP of system.

**Authority Setup**(GP)→SK,PK: Taking GP as input, each authority generates a secret key SK and public key PK corresponding to the attribute belonging to the authority. This algorithm is executed at each attribute authority, i.e, hospital AA, and university AA.

**Encrypt**$(M_{i},W,PK)\to CT$: Taking PK and an access policy $W=[W_{1}$ OR $W_{2}$ OR…OR $W_{p^{\prime }}]$ as input; the algorithm encrypts each message $M_{i}$ correspondingly with attribute set $W_{i}$ of policy for $1\leq i\leq p^{\prime }$ to output a ciphertext CT. This algorithm is executed by patient.

**KeyGen**$(GID,PK,L,x,SK)\to K_{x,GID}$: A user with a global identifier GID has an attribute set *L*, where x∈L. This algorithm generates a key $K_{x,GID}$ corresponding to an attribute *x* and identity GID of user. This algorithm is executed at each AA.

**Decrypt**$(CT,PK,L,K_{x,GID})\to M_{i}$: Taking the ciphertext and user attribute key set *L* as input, the algorithm outputs a message $M_{i}$ corresponding to attribute set $W_{i}$ of policy *W*; if L⊧W. This algorithm is executed by the user’s with contextual attributes like doctor, nurse, professor, and insurance agent.

**Policy Update**$(PK,S_{owner},W,W^{\prime })\to U$: The algorithm outputs the update parameter U by taking as input the original policy *W*, update in policy $W^{\prime }$, PK and owner secret $S_{owner}$ embedded in ciphertext during encryption. This algorithm is executed by patient for updating the access control policy.

**Ciphertext Update**$(CT,U)\to CT^{\prime }$: The algorithm outputs the updated ciphertext $CT^{\prime }$ by taking as input the original CT and ciphertext update U parameter. This algorithm is executed at cloud.

### 5.2. Formalized Security Model

In this section, we present the security model for proving our cryptosystem. The detailed security proof is in Section 7.1. We consider the following indistinguishability game under selective chosen-plaintext-attacks (IND−sCPA) between an adversary A and challenger C for PC-MM-CP-ABE-EPU scheme. **Init**

Adversary specifies and sends a challenge access policy structure $W^{*}$ to C.

**Setup**C runs the global and authority setup algorithms to generate the global parameters GP and secret/public keys of attributes. It then gives the public keys and GP to A.

**Phase 1**A queries for the secret keys by providing an attribute list *L* and identities GID. C replies with secret keys if *L* does not satisfy $W^{*}$.

**Challenge**A specifies two distinct equal length messages ($M_{0,i}\neq M_{1,i}$) correspondingly for each attribute set $W_{i}^{*}$ in policy $W^{*}$ and an update parameter $U^{*}$. In response, C chooses bit β={0,1} at random, computes $CT^{*}$ = **Encrypt**$\left(M_{\beta ,i},W^{*},GP,PK\right)$, and sends it to A if $U^{*}=\vartheta$. Otherwise, it sends $CT^{\prime }$ = **Update**$(CT^{*},U^{*})$ to A.

**Phase 2**A continues to query for secret keys under the same constraint that the access structure $W^{*}$ should not be violated.

**Guess**A outputs a guess $\beta^{\prime }$ for β and wins the game if $\beta^{\prime }=\beta$. Advantage of A in winning the IND-sCPA game is

$$Adv_{PC-MM-CP-ABE-EPU}^{IND-sCPA}\left(A\right)=\left|Pr\left[\beta^{\prime }=\beta \right]-\frac{1}{2}\right|$$

## 6. Proposed Scheme

In this section, firstly we discuss the intuition behind the multi message CP-ABE and hierarchical access control provision in Section 6.1, and later in Section 6.2 we detail a concrete cryptographic construction for encforcing patient centric access control.

### 6.1. Methodology

To illustrate our idea, we begin with a simple example of monotone policy and then further extend it to define our intuition. A monotone access policy in its Disjunctive Normal Form (DNF) representation itself contains the individual AND (∧) based access structures. Hence, combination of AND (∧) based access policy [35,36] by placing OR between them itself leads to an expressive monotone access policy [13]. Consider a patient-centric policy as *W* = (Hospital-1 ∧ Doctor) OR (Hospital-1 ∧ Nurse) OR (University-1 ∧ Professor) OR (University-1 ∧ Student) OR (Insurance company-1 ∧ Insurance-agent). This policy $W=[W_{1}$ OR $W_{2}$ OR $W_{3}$ OR $W_{4}$ OR $W_{5}]$ is comprised of 5 attribute sets namely, $W_{1}$ = (Hospital-1, Doctor), $W_{2}$ = (Hospital-1, Nurse), $W_{3}$ = (University-1, Professor), $W_{4}$ = (University-1, Student), $W_{5}$ = (Insurance company-1, Insurance-agent). Any user conforming to the policy (L⊧W) needs to satisfy at least one attribute set $W_{i}$ of policy for i={1,2,3,4,5}. A user attribute set *L* satisfies policy L⊧W if $W_{i}\subset L$ [37], i.e., any attribute set $W_{i}$ of policy should be the subset of user attribute set *L*.

For further elaboration, Table 3 indicates five arbitrary user’s along with their attribute sets *L* indicating whether or not they satisfy the policy *W*.

Suppose after sometime, the patient updates the policy *W* into $W^{u}$ as $W^{u}$ = (Hospital-1 ∧ Doctor) OR (Hospital-1 ∧ ENT ∧ Nurse) OR (University-2 ∧ Professor) OR (University-2 ∧ Student). After policy update, some contextual user’s who were previously granted data access cannot access data after policy update.

For updating the policy, the data owner generates an update parameter U comprising of attributes that needs to be added or revocated from an existing attribute set $W_{i}$ of policy W. Secret $t_{i}$ values corresponding to $W_{i}$ which were embedded in CT are utilized by data owner for generating the update parameter. Hence, an owner needs to keep a record of secret $t_{i}$ values for policy updates in the future. The update U is sent to the server, and it runs the update algorithm for updating CT corresponding to new policy. However, the server cannot exploit both U and CT to get more information. Moreover, if any user satisfying the policy prior to its update has not decrypted CT; so he will also not be able to decrypt it after update if now he does not satisfy the new policy.

In traditional CP-ABE schemes [4,13,35,36] a single message is embedded into a ciphertext for all the attribute sets $W_{i}$ of policy. So, all users satisfying any individual $W_{i}$ of policy leads to the same secret “*s*” re-construction, and hence have access to the same data. However, this cannot be adopted in the provision of hierarchal access control because there is a need to embed multiple messages in a single ciphertext over policy. To cater, we embed multiple secrets $t_{i}$ corresponding to attribute sets $W_{i}$ of policy *W* for encrypting multiple messages. This enables users satisfying any different $W_{i}$ to have access to different granularities of data. For enforcing hierarchal access control, data needs to be divided logically into chunks $m_{1},m_{2},\ldots ,m_{p^{\prime }}$ and each chunk encrypted with hierarchically derived key [15,38,39,40]. In this technique, several chunk keys are obtained from parent-node key such that key derivation follows the top-down (1-way) approach, i.e., from the parent node to descendant child-nodes. We detail the hash based key derivation [15].

The key $k_{i}$ generation corresponding to the $i^{th}$ level of hierarchy is proceeded as:

$$k_{i}=H(k_{i+1}||i)\;for\;i=p^{\prime }-1,\ldots ,3,2,1$$

For generating *p* number of chunk keys at the same hierarchical level from key *k* is proceeded as:

$$k_{i}=H(k||i)\;for\;i=1,2,3,\ldots ,p^{\prime }$$

where *H* is a standard one-way hash function. Table 3 depicts the granular data access control that doctor and professor have access to all data. However, student and nurse are restricted to a limited proportion of logical data as specified by the patient.

### 6.2. PC-MM-CP-ABE-EPU

In this subsection, we present our proposed Patient-Centric Multi Message Ciphertext Policy-Attribute Based Encryption with Efficient Policy Update (PC-MM-CP-ABE-EPU). We assume that there exist *u* attributes in the universe. Formally, in encryption, the public keys of involved attributes are aggregated to form a single attribute. Similarly, the decryption process includes an aggregation of user attribute keys satisfying policy. Hence, the construction leads to constant computational cost in encryption and decryption and is independent of the number of attributes. Moreover, each user has a unique identity by mapping its GID to a random group element, thereby restricting users to collude their attribute keys. To construct multi-message CP-ABE in a single ciphertext over policy, multiple secrets corresponding to different attribute sets of policy are embedded in ciphertext; in-contrast to a single secret for traditional CP-ABE schemes. Moreover, data owner acts as an enforcer of policy updates, while the server updates the ciphertext. For policy update, i.e., the addition or revocation of attributes; data owner generates the update parameter requiring only 2 exponential group operations on its side, while a single multiplication of group elements is performed at the server side.

The algorithms of the proposed scheme is defined as:

**Global Setup**(λ)→GP: In global setup, a bilinear group *G* of prime order *p* is chosen. Global parameters are set to *p*, *g*, e(g,g) and *H*; where *g* is a generator of group *G* and *H* is a hash function that maps global identities GID to elements in *G*.

**Authority Setup**(GP)→SK,PK: For every attribute *x* that belongs to an authority, it chooses two random values $a_{x},b_{x}\in Z_{p}$. It sets secret key as $SK=\{a_{x},b_{x}\in Z_{p}$} and publishes public key as $PK=\{g^{-a_{x}},e{\left(g,g\right)}^{b_{x}}\}$.

**Encrypt**$(M_{i},W,PK)\to CT$: Data owner defines an access policy $W=[W_{1}$ OR $W_{2}$ OR…OR $W_{p^{\prime }}]$, where $W_{i}$ for i=1 to $p^{\prime }$ corresponds to an attribute set in policy. All the attributes with in an attribute set $W_{i}$ have an AND operation between them stating the significance that all of them must be present for satisfaction of policy. For enforcing hierarchal (different) access control corresponding to different $W_{i}$ of policy, data owner divides the data logically into chunks, where each data chunk $M_{i}$ is encrypted with hierarchically derived key $k_{i}$ for $1\leq i\leq p^{\prime }$ as illustrated in Section 6.1.

For notational simplicity, we represent keys $k_{i}$ with messages $M_{i}$ in the rest of the paper. For each message $M_{i}$ corresponding to each attribute set $W_{i}$ of policy it chooses a random owner secret $S_{owner}=t_{i}\in Z_{p}$. After then, it aggregates the PK of attributes from relevant authorities belonging to each $W_{i}$ and computes the ciphertext as:

$$C_{1,i}=g^{t_{i}},C_{2,i}={\left(\prod_{x\in W_{i}}g^{-a_{x}}\right)}^{t_{i}},C_{3,i}=M_{i}*{\left(\prod_{x\in W_{i}}e{\left(g,g\right)}^{b_{x}}\right)}^{t_{i}}$$

The owner then sends $CT=\{W,C_{1,i},C_{2,i},C_{3,i}\}$ for $1\leq i\leq p^{\prime }$ to the server.

**KeyGen**$(GID,PK,x,SK)\to K_{x,GID}$: To create a key for user GID corresponding to an attribute *x* of authority, the authority computes:

$$K_{x,GID}=g^{b_{x}}\cdot H{\left(GID\right)}^{a_{x}}$$

We remark that any user with a global identifier GID has an attribute set *L*, where x∈L, and a user can have more than one attributes keys based on his attributes in set *L*.

**Decrypt**$(CT,PK,L,K_{x,GID})\to M_{i}$: If user attribute set *L* satisfies the condition $W_{i}\subset L$ for an attribute set $W_{i}$ in policy *W*; then he satisfies the policy L⊧W and proceeds by calculating the aggregated key as $K=\prod_{x\in W_{i}}K_{x,GID}$. To retrieve plaintext message $M_{i}$ for corresponding $W_{i}$, user computes:

$$C_{3,i}/e\left(H\left(GID\right),C_{2,i}\right)*e\left(K,C_{1,i}\right)=M_{i}$$

**Policy Update**$(PK,S_{owner},W,W^{\prime })\to U$: For policy update, it takes $W^{\prime }=[W_{1}^{\prime }$ OR $W_{2}^{\prime }$ OR…OR $W_{p^{\prime }}^{\prime }]$; where $W_{i}^{\prime }$ contains the list of attributes to be added or revocated from the particular attribute set $W_{i}$ in original policy *W*. Intuitively, the addition of attributes is performed when $W_{i}^{\prime }\cap W_{i}=\vartheta$ resulting in an updated attribute set policy as $W_{i}^{u}=W_{i}^{\prime }\cup W_{i}$. Similarly, for attribute revocation the condition $W_{i}^{\prime }\subset W_{i}$ needs to be satisfied resulting in an updated policy as $W_{i}^{u}=W_{i}\setminus W_{i}^{\prime }$ correspondingly for a particular attribute set in *W* for $i=\{1,2,\ldots ,p^{\prime }\}$. Moreover, it takes the product of public keys PK and sets the parameters

$$u_{1,i}={\left(\prod_{x\in W_{i}^{\prime }}g^{-a_{x}}\right)}^{o}\;,\;u_{2,i}={\left(\prod_{x\in W_{i}^{\prime }}e{\left(g,g\right)}^{b_{x}}\right)}^{o}$$

correspondingly for attributes addition or revocation from an existing attribute set $W_{i}$. For performing the addition of attributes data owner sets *o* to $S_{owner}=t_{i}$; while for revocation of attributes it sets *o* as $-S_{owner}=-t_{i}$. Data owner then sets the update parameter as $U=\{W_{i}^{u},u_{1,i},u_{2,i}\}$ where $W_{i}^{u}$ contains the updated list of attributes after addition or revocation of attributes from the particular attribute set $W_{i}$ in original policy *W*.

**Ciphertext Update**$(CT,U)\to CT^{\prime }$: This algorithm takes as input the original CT and update parameter $U=\{W_{i}^{u},u_{1,i},u_{2,i}\}$. For policy update, it takes the parameters $u_{1,i},u_{2,i}$ and multiply them correspondingly by original ciphertext CT components $C_{2,i},C_{3,i}$ for a particular $W_{i}$ for $i=\{1,2,\ldots ,p^{\prime }\}$ as

$$C_{2,i}^{\prime }=C_{2,i}\cdot u_{1,i},C_{3,i}^{\prime }=C_{3,i}\cdot u_{2,i}$$

to obtain the updated ciphertext $CT^{\prime }=\left\{W^{u},C_{1,i},C_{2,i}^{\prime },C_{3,i}^{\prime }\right\}$ for the updated policy. Observe that, the distribution of $CT^{\prime }$ is similar to CT.

### 6.3. Correctness Decryption

In this subsection, we prove the correctness of the decryption algorithm.

$$\frac{C_{3,i}}{e\left(H\left(GID\right),C_{2,i}\right)*e\left(K,C_{1,i}\right)}$$

$$\frac{M_{i}*{\left(\prod_{x\in W_{i}}e{\left(g,g\right)}^{b_{x}}\right)}^{t_{i}}}{e\left(H\left(GID\right),{\left(\prod_{x\in W_{i}}g^{-a_{x}}\right)}^{t_{i}}\right)*e\left(\prod_{x\in W_{i}}g^{b_{x}}H{\left(GID\right)}^{a_{x}},g^{t_{i}}\right)}=M_{i}$$

### 6.4. Correctness Policy Update

For attribute’s addition, $o=t_{i}$, hence updated policy is $W_{i}^{u}=W_{i}^{\prime }\cup W_{i}$, and the shares of newly added attributes is aggregated to already present attributes to transform the final ciphertext policy as: $C_{2,i}^{\prime }=\prod_{x\in W_{i}^{u}}g^{-a_{x}}{)}^{t_{i}},C_{3,i}^{\prime }=M_{i}*{\left(\prod_{x\in W_{i}^{u}}e{\left(g,g\right)}^{b_{x}}\right)}^{t_{i}}$. For attribute’s revocation, $o=-t_{i}$, hence updated policy is $W_{i}^{u}=W_{i}\setminus W_{i}^{\prime }$, and the shares of revocated attributes are cancelled out (due to negative/negation operation) from already present attributes to transform the final ciphertext policy as: $C_{2,i}^{\prime }=\prod_{x\in W_{i}^{u}}g^{-a_{x}}{)}^{t_{i}},C_{3,i}^{\prime }\;=\;M_{i}\;*\;{\left(\prod_{x\in W_{i}^{u}}e{\left(g,g\right)}^{b_{x}}\right)}^{t_{i}}$. We remark that the updated ciphertext is similar to (in form) and indistinguishable from the original ciphertext in policy.

$$C_{2,i}^{\prime }=C_{2,i}\cdot u_{1,i}={\left(\prod_{x\in W_{i}}g^{-a_{x}}\right)}^{t_{i}}\cdot {\left(\prod_{x\in W_{i}^{\prime }}g^{-a_{x}}\right)}^{o}$$

$$C_{3,i}^{\prime }=C_{3,i}\cdot u_{2,i}=M_{i}*{\left(\prod_{x\in W_{i}}e{\left(g,g\right)}^{b_{x}}\right)}^{t_{i}}\cdot {\left(\prod_{x\in W_{i}^{\prime }}e{\left(g,g\right)}^{b_{x}}\right)}^{o}$$

## 7. Analysis and Discussion

Security and performance are the two major metrics that need to be evaluated from the prospect of any secure and efficient cryptographic scheme. This section, therefore, discusses the security and performance of the proposed scheme with the help of security proof and experimentations.

### 7.1. Security

With reference to the security model presented in Section 5.2, here we prove the following theorem to exhibit the security of the proposed scheme.

**Theorem** **1.** *We show that the proposed PC-MM-CP-ABE-EPU scheme is selectively secure under chosen-plaintext-attacks (IND-sCPA) by a game played between an adversary A and challenger C as described in Section 5.2.*

**Proof of Theorem 1.**  We suppose the existence of an adversary A to break the proposed construction with a non-negligible advantage. We thus build a simulator B to interact with A in the IND-sCPA game; where B plays the role of challenger, and has an advantage ϵ to solve q-BDHE problem in group *G*. Suppose challenger inputs a q-BDHE instance $\left(g,h=g^{s},y_{g,\alpha ,q},T\right)$ for a single encrypted message, where $y_{g,\alpha ,q}=\left(g,g_{1},g_{2},\ldots ,g_{q},g_{q+2},\ldots ,g_{2q}\right)$ for $g_{i}=g^{\alpha^{i}}$ where $\alpha \in Z_{p}^{*}$.

We consider a slight modification in q-BDHE instance where $h_{i}=g^{t_{i}}$ for different attribute sets $W_{i}$ in policy *W* instead of $h=g^{s}$ for a single challenged attribute set in policy. Now, challenger inputs a modified q-BDHE instance $\left(g,h_{i}=g^{t_{i}},y_{g,\alpha ,q},T_{i}\right)$. Infact, in the proof B encrypts messages $M_{\beta ,i}$ for all the different attributes sets $W_{i}^{*}$ in the challenged access structure $W^{*}$.

**Init**A specifies and sends a challenge access structure $W^{*}=\left[W_{1},W_{2},\ldots ,W_{p^{\prime }}\right]$ to B.

**Setup**B selects randomly $j^{*}\in_{R}I_{X_{i}}$ for every attribute set $W_{i}^{*}$, where $I_{X_{i}}=\left\{1,2,\ldots ,m\right\}$ is the index of the attributes appearing in $W_{i}^{*}$. B picks $a_{j^{*}},c_{j^{*}}\in_{R}Z_{p}$ for each $j^{*}\in_{R}I_{X_{i}}$, and $a_{j},c_{j}\in_{R}Z_{p}$ for k={1,2,…,n}. We remind here, that all attribute authorities are working in a decentralized fashion, and all attribute authorities work in a similar fashion for parameters generation. Here, challenger acts on behalf of authorities to generate parameters. For setting the public parameters correspondingly for each attribute authority, B proceeds as:

1. if $j=j^{*}$, then $\left(A_{j},B_{j}\right)$ = $(g^{-a_{j^{*}}}$ $\prod_{j\in I_{X_{i}}-\left\{j^{*}\right\}}$ $g_{q+1-j}$, $e{\left(g,g\right)}^{c_{j^{*}}}$$e{\left(g,g\right)}^{\alpha^{q+1}})$
2. if $j\in I_{X_{i}}-\left\{j^{*}\right\}$, then $\left(A_{j},B_{j}\right)$ = $(g^{-a_{j}}g_{q+1-j}^{-1}$, $e{\left(g,g\right)}^{c_{j}})$
3. if $j\notin I_{X_{i}}$, then $\left(A_{j},B_{j}\right)$ = $\left(g^{-a_{j}},e{\left(g,g\right)}^{b_{j}}\right)$

B then sends public key $\left(A_{j},B_{j}\right)$ to A for the all attributes belonging to different authorities.

**Phase 1**A submits the several key queries corresponding to particular GID and an attributes set *L* of his choice. Generally, for such queries we assume that there should be at-least one attribute $att_{x}\in L$ for which the key query cannot be made, such that $L\neq W_{i}^{*}$. Particularly, the constraint is that the set *L* should not satisfy any of attribute sets $W_{i}^{*}$ in policy. In response, B responds by selecting $z\in_{R}Z_{p}$ and sets $H\left(GID\right)=g_{j}g^{z}$. Further, it sets the decryption keys correspondingly for attributes belonging to different authorities. For each authority, B sets the keys as:

1. if $j=j^{*}$, then $K_{j,GID}={\left(g_{j}\right)}^{a_{j}}g^{c_{j}}(\prod_{j\in I_{X_{i}}-\left\{j^{*}\right\}}$$g_{q+1-j+j^{*}}^{-1})$${\left(B_{j}\right)}^{-z}$
2. if $j\in I_{X_{i}}-\left\{j^{*}\right\}$, then $K_{j,GID}={\left(g_{j}\right)}^{a_{j}}$$g^{c_{j}}$$g_{q+1-j+j^{*}}$${\left(B_{j}\right)}^{-z}$
3. if $j\notin I_{X_{i}}$, then $K_{j,GID}={\left(g_{j}g^{z}\right)}^{a_{j}}g^{b_{j}}$

Finally, B returns the keys $K_{j,GID}$ to A for particular identities GID and user queried attribute set *L*.

**Challenge** In this phase, A submits two distinct equal length messages ($M_{0,i}\neq M_{1,i}$) correspondingly for each attribute set $W_{i}^{*}$ specified in policy $W^{*}$ and an update parameter $U^{*}$ to B. Simulator responds by setting $a_{I,i}$ and $c_{I,i}$; as $a_{I,i}=\sum_{j=1}^{m}a_{j}$ and $c_{I,i}=\sum_{j=1}^{m}c_{j}$. Then, B chooses $\beta \in_{R}\left\{0,1\right\}$ and calculates the ciphertext $CT^{*}$ for the entire policy $W^{*}$. Moreover, accordingly to update parameter $U^{*}$, it updates and sets the ciphertext $CT^{\prime }$ as:

$C_{1,i}^{\prime }=h_{i}=g^{t_{i}}$,
$C_{2,i}^{\prime }={\left(\prod_{j\in W_{i}}g^{-a_{j}}\right)}^{t_{i}}=h_{i}^{-a_{I,i}^{\prime }}$,
$C_{3,i}^{\prime }=M_{\beta ,i}\cdot {\left(\prod_{j\in W_{i}}e{\left(g,g\right)}^{b_{j}}\right)}^{t_{i}}$$=M_{\beta ,i}\cdot e\left(g_{q+1},h_{i}\right)\cdot e{\left(g,h_{i}\right)}^{c_{I,i}^{\prime }}$

B sends the ciphertext $CT^{\prime }$ to A.

**Phase 2** Similar to Phase 1.

**Guess**A outputs a guess $\beta^{\prime }$ for β. For $\beta^{\prime }=\beta$, B outputs $v^{\prime }=0$, and vice-versa for other case.

**Probability Analysis** Given a q-BDHE instance $\left(g,h_{i}=g^{t_{i}},y_{g,\alpha ,q},T_{i}\right)$ to B, and an A breaks our PC-MM-CP-ABE-EPU with advantage ϵ. Then we present the analysis of two cases below.

Case 1 ($U^{*}=\vartheta$) In this case when there is no policy update, B sets the ciphertext $CT^{*}$ as:

$C_{1,i}^{*}$$=h_{i}=g^{t_{i}}$
$C_{2,i}^{*}$$={\left(\prod_{j\in W_{i}}g^{-a_{j}}\right)}^{t_{i}}={\left(g^{-a_{j^{*}}}\prod_{j\in I_{X_{i}}-\left\{j^{*}\right\}}g_{q+1-j}\cdot \prod_{j\in I_{X_{i}}-\left\{j^{*}\right\}}g^{-a_{j}}g_{q+1-j}^{-1}\right)}^{t_{i}}$$=h_{i}^{-a_{I,i}}$
$C_{3,i}^{*}$$=M_{\beta ,i}\cdot {\left(\prod_{j\in W_{i}}e{\left(g,g\right)}^{b_{j}}\right)}^{t_{i}}$$=M_{\beta ,i}\cdot e{\left(g,g\right)}^{c_{j^{*}}+\alpha^{q+1}}$$\prod_{j\in I_{X_{i}}-\left\{j^{*}\right\}}$$e{\left(g,g\right)}^{c_{j}}{)}^{t_{i}}$
  $\;=M_{\beta ,i}\cdot e\left(g_{q+1},h_{i}\right)\cdot e{\left(g,h_{i}\right)}^{c_{I,i}}$

We note that ciphertext $CT^{*}=\left\{C_{1,i}^{*},C_{2,i}^{*},C_{3,i}^{*}\right\}$ is a valid encryption of message $M_{\beta ,i}$ if $T_{i}=e\left(g_{q+1},h_{i}\right)$; otherwise, if it a random group element, i.e., $T_{i}\in G_{T}$, then $CT^{*}$ is independent of β in A view. For $v^{\prime }=0$, the ciphertext $CT^{*}$ is valid and $T_{i}$ is set as $e(g_{q+1},h_{i})$. A can guess correct $\beta^{\prime }$ with a non-negligible advantage defined by $Pr\left[\beta^{\prime }=\beta |v^{\prime }=0\right]=\frac{1}{2}+\epsilon$. For $v^{\prime }=1$, $T_{i}\in G_{T}$, $CT^{*}$ cannot be identified and we have $Pr\left[\beta^{\prime }\neq \beta |v^{\prime }=1\right]=\frac{1}{2}$. From the analysis, the probability with which B succeeds in breaking the q-BDHE assumption is: $\frac{1}{2}Pr\left[\beta^{\prime }=\beta |v^{\prime }=0\right]+\frac{1}{2}Pr\left[\beta^{\prime }\neq \beta |v^{\prime }=1\right]=\frac{1}{2}+\frac{\epsilon }{2}$.

Case 2 ($U^{*}=\left(att_{1},att_{2},\ldots ,m^{\prime }\right)\neq \vartheta$) In the case, when the A has requested for a policy update, B proceeds by first calculating the $CT^{*}$ as above.

$C_{1,i}^{*}$$=h_{i}=g^{t_{i}}$
$C_{2,i}^{*}$$={\left(\prod_{j\in W_{i}}g^{-a_{j}}\right)}^{t_{i}}={\left(g^{-a_{j^{*}}}\prod_{j\in I_{X_{i}}-\left\{j^{*}\right\}}g_{q+1-j}\cdot \prod_{j\in I_{X_{i}}-\left\{j^{*}\right\}}g^{-a_{j}}g_{q+1-j}^{-1}\right)}^{t_{i}}$$=h_{i}^{-a_{I,i}}$
$C_{3,i}^{*}$$=M_{\beta ,i}\cdot {\left(\prod_{j\in W_{i}}e{\left(g,g\right)}^{b_{j}}\right)}^{t_{i}}$$=M_{\beta ,i}\cdot e{\left(g,g\right)}^{c_{j^{*}}+\alpha^{q+1}}$$\prod_{j\in I_{X_{i}}-\left\{j^{*}\right\}}$$e{\left(g,g\right)}^{c_{j}}{)}^{t_{i}}$
  $\;=M_{\beta ,i}\cdot e\left(g_{q+1},h_{i}\right)\cdot e{\left(g,h_{i}\right)}^{c_{I,i}}$

After then, for addition or revocation of attributes specified by A in $U^{*}$; B runs the Update($CT^{*},U^{*})\to CT^{\prime }$ algorithm to update the ciphertext. B proceeds by updating the $a_{I,i}$ and $c_{I,i}$ to $a_{I,i}^{\prime }$ and $c_{I,i}^{\prime }$ respectively because of addition and revocation of attributes from particular attribute sets in $CT^{*}$. Precisely, $a_{I,i}^{\prime }=\sum_{j=1}^{m^{\prime }}a_{j}$ and $c_{I,i}^{\prime }=\sum_{j=1}^{m^{\prime }}c_{j}$ and the distribution of the $CT^{\prime }$ is identical to $CT^{*}$. Finally, the set values of $CT^{\prime }$ is

$C_{1,i}^{\prime }$ = $g^{t_{i}}$
$C_{2,i}^{\prime }$ = $h_{i}^{-a_{I,i}^{\prime }}$
$C_{3,i}^{\prime }$ = $M_{\beta ,i}\cdot e\left(g_{q+1},h_{i}\right)\cdot e{\left(g,h_{i}\right)}^{c_{I,i}^{\prime }}$

We note that ciphertext $CT^{\prime }=\left\{C_{1,i}^{\prime },C_{2,i}^{\prime },C_{3,i}^{\prime }\right\}$ is a valid encryption of message $M_{\beta ,i}$ if $T_{i}=e\left(g_{q+1},h_{i}\right)$; otherwise, if it a random group element, i.e., $T_{i}\in G_{T}$, then $CT^{\prime }$ is independent of β in A view. For $v^{\prime }=0$, the ciphertext $CT^{\prime }$ is valid and $T_{i}$ is set as $e(g_{q+1},h_{i})$. A can guess correct $\beta^{\prime }$ with a non-negligible advantage defined by $Pr\left[\beta^{\prime }=\beta |v^{\prime }=0\right]=\frac{1}{2}+\epsilon$. For $v^{\prime }=1$, $T_{i}\in G_{T}$, $CT^{\prime }$ cannot be identified and we have $Pr\left[\beta^{\prime }\neq \beta |v^{\prime }=1\right]=\frac{1}{2}$. From the analysis, the probability with which B succeeds in breaking the q-BDHE assumption is: $\frac{1}{2}Pr\left[\beta^{\prime }=\beta |v^{\prime }=0\right]+\frac{1}{2}Pr\left[\beta^{\prime }\neq \beta |v^{\prime }=1\right]=\frac{1}{2}+\frac{\epsilon }{2}$.

**Note:** There is an assumption that attributes are not repeated in the policy $W^{*}$. □

### 7.2. Performance Analysis

To demonstrate the performance of the proposed scheme, we firstly compare algorithmically our proposed scheme with the existing healthcare CP-ABE schemes. Moreover, to evaluate the effectiveness of the proposed scheme, the computation time of encryption, decryption, and policy update algorithms is evaluated by varying the number of attributes in the policy. In addition, for the effectiveness of model in real-time scenarios, a shimmer sensor has been employed, whose results are discussed below.

#### 7.2.1. Algorithmic Complexity Analysis

Here, we give the performance analysis of our proposed scheme taking into consideration the existing relevant schemes. We remark that the encryption, decryption operations, and ciphertext size are the main factors affecting the communication and computation cost of the overall system. The user key generation is a one time process, hence it does not contribute significantly. Table 4 gives a comparison of our proposed scheme with existing multi message CP-ABE schemes [15,16], and healthcare centric CP-ABE schemes, [5,6,7,8]. None of the existing schemes facilitates the patient with defining access control policy. As seen from the Table 4, the encryption cost of the proposed scheme is constant with 3 exponential operations for an attribute set $W_{i}$ in policy. The decryption process comprises 2 pairing operations. Moreover, for the proposed scheme both encryption, decryption operations, and ciphertext size are independent of the number of attributes *n* in the policy; in-contrast to its dependence on attributes in other relevant existing schemes.

We compare the policy update feature of our scheme with Jiang et al. [26], Belguith et al., and [28] Li et al. [27] based on several parameters as seen from Table 5. The policy update operation is performed at the data owner/patient side, while the ciphertext update operation is performed at the server side. The encryption, ciphertext size, policy update, and ciphertext update costs is a function of $p^{\prime }$ in the proposed scheme, where $p^{\prime }$ is the number of attributes sets $W_{i}$ in the policy. However, for [26,27,28] the costs varies based on variables *u*, *n* and *t*, where *u* is the total number of attributes in universe, *n* is the number of attributes in access structure, *t* is the number of revocated attributes from ciphertext. Typically *u* and *n* generally have large values like *u* = 1000 and *n* = 30–100, and $p^{\prime }$ is smaller like 5–15; hence, the fact reveals that costs for the proposed scheme are fairly less incontrast to [26,27,28]. This fact has been demonstrated with experimental results below.

#### 7.2.2. Computational Complexity Analysis

In this section, we demonstrate our proof-of-concept prototype by implementing our proposed solution using a client-server architecture. All our simulation results presented here are carried on a Ubuntu 14.04 virtual machine with 2GB allocated Ram on a Dell Inspiron i3-6006U CPU@2GHz laptop with 8GB RAM. To test the feasibility of the proposed scheme, we have used a physiological health sensor: Shimmer3 motion (IMU) coupled with biophysical units. Table 6 highlights the different data streams that can be gathered through the Shimmer sensor and their sampling rate. The sampling rate of 128 Hz by GSR sensor indicates it generates 128 samples per second. However, the parameter values generated by the sensors differ, like GSR generates only 1 parameter value per sample. In-contrast, the Gyroscope and ECG sensors generate 3 and 4 parameter values per sample respectively.

The shimmer sensor was worn by one of the participant and the physiological data stream was gathered for testing the feasibility of the scheme presented in this paper. We simulated the CP-ABE based policy specification/update results on a pairing based crypto library Charm [41], and the physiological sensors data encryption using Advanced Encryption Standard (AES) by employing Cipher Block Chaining (CBC) mode in pycryptodomex library [42]. We employed “SS512” symmetric curve with a base field of 512 bit to implement CP-ABE based pairing operations in Charm. The time presented here is the average over ten iterations in Charm and pycryptodomex libraries.

**Figure 3:**
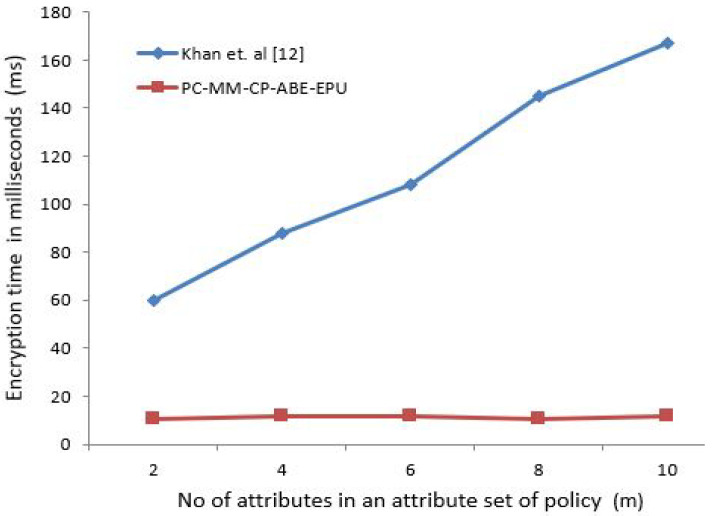
Patient Policy Encryption Time (ms) for # of attributes.

**Figure 4:**
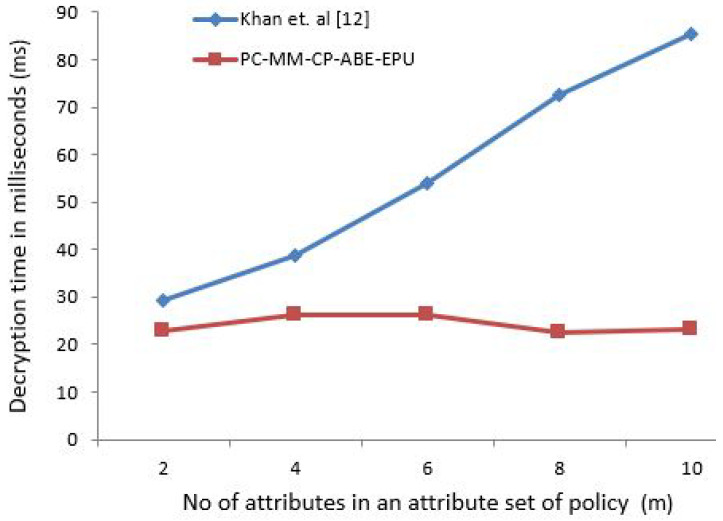
Contextual User’s Conforming Policy for data access.

**Figure 5:**
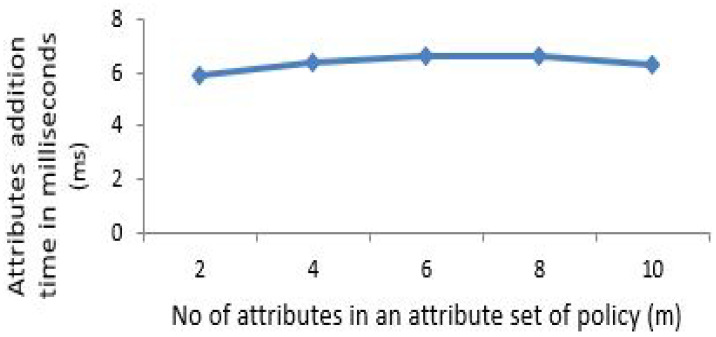
Attributes addition by patient for policy update.

**Figure 6:**
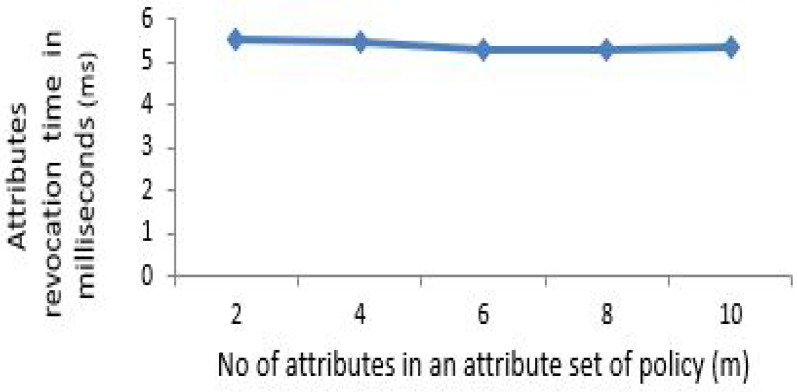
Attributes revocation by patient for policy update.

The x-coordinate depicts the number of attributes in an attribute set of policy. To elaborate that the computation and policy update cost of proposed scheme is almost constant for any number of attributes to be specified under policy, the number of attributes needs to be gradually increased to depict this effect. For Figure 3, Figure 4, Figure 5 and Figure 6 horizontal axis shows the gradual increase of attributes to depict the effect.

Figure 3 shows the encryption time in milliseconds (ms) for the number of attributes specified by the patient. Similarly, Figure 4 shows the decryption time in milliseconds (ms) for user’s with contextual attributes, i.e., doctor, nurse, and professor. As our proposed scheme is independent of the involvement of the attributes in encryption and decryption operations, hence the time is almost constant in contrast to [16] as seen from Figure 3 and Figure 4.

For updating the policy, the generation of update parameter U involves an exponential operation and several multiplications depending on the number of attributes addition or revocation for our proposed scheme. Figure 5 indicates the time in (ms) taken by the patient for adding additional number of attributes to an existing policy. Similarly, Figure 6 shows it for the case of attributes revocation by patient from an existing defined policy. Figure 5 and Figure 6 exhibit a fractional change in time of around 1 ms (almost constant) which incurred due to multiplication of group elements while increasing attributes from 2 to 10.

Any contextual user satisfying the policy will have access to data, this literally means that contextual user will have access to AES data encryption/decryption key with which the data owner has performed encryption. Considering the role of patient, we encrypted the data generated by the shimmer sensor-GSR, Gyroscope, and ECG with AES in CBC mode for 1 s time span. Table 7 shows the average encryption, decryption time in (ms) for the selected sensors. As the sampling rate and parameters per sample vary for all three sensors, hence the ciphertext size is different for all of the sensors as presented in Table 7.

The policy specification/update or its conformance is normally a one time process, and takes similar amount of time as seen from Figure 3, Figure 4, Figure 5 and Figure 6 in comparison to data encryption timings as seen from Table 7. For real-time health care data acquisition, monitoring, analysis, and diagnosis; the policy specification and data encryption time should be less, so that an uninterrupted synchronous data transmission can be achieved between both parties. This fact can also be validated from the worst-case ECG sensor encryption time of 68 ms which is less than 1 s. We affirm that the proposed scheme performance is independent of the sensor used to generate the data.

## 8. Conclusions and Future Work

To address simultaneously the challenges of enforcing hierarchal access control and providing dynamic access privileges for healthcare, in this paper, we have proposed the notion of an efficient patient centric multi message CP-ABE with policy update. The proposed scheme can encrypt multiple messages to ensure access control for hierarchal groups of users resulting in users having access to different granularities of the same data. Moreover, the data owner can dynamically enforce addition or revocation of attributes from policy. Performance analysis of the scheme depicts that computation and communication costs incurred by the construction are almost constant; in contrast to depending on number of involved attributes in policy. Moreover, it is proven to be selectively secure under q-BDHE assumption in random oracle model. In future, we will extend the proof-of-concept prototype and integrate it with a public cloud platform.

## Figures and Tables

**Table 1:** Applications of CP-ABE schemes in Healthcare.

Scheme	AA	Security	Feature	PU
[5]	Single	SS-SM under decisional q parallel BDHE	online-offline encryption for resource constrained client	No
[6]	Single	FS-SM under 3 assumptions in composite order group	doctor centric key delegation decryption mechanism	No
[7]	Single	SS-SM under decisional q parallel BDHE, c-BDHE	online-offline attribute based proxy re-encryption	No
[8]	Single	FS-GGM	searchable trapdoor CP-ABE	No
[9]	Single	N/A	searchable trapdoor CP-ABE	No
This Work	Multiple	SS-ROM under q-BDHE	patient-centric CP-ABE with policy update & const costs	Yes

SS: Selective Secure, FS: Fully Secure, SM: Standard Model, GGM: Generic Group Model, BDHE: Bilinear Diffie Hellman Exponent Assumption, PU: Policy Update Feature of CP-ABE, ROM: Random Oracle Model.

**Table 2:** Notations.

Symbol	Description
*p*	Bilinear group order
$p^{\prime }$	Maximum number of attribute sets
$W_{i}$	A particular attribute set of policy
*m*	Number of attributes within an attribute set $W_{i}$
*n*	Total number of attributes in the access structure
*W*	Ciphertext Policy comprising of several $W_{i}$
*L*	User attribute set consisting of various attributes
L⊧W	User attribute set satisfying policy
L≠W	User attribute set not satisfying policy
$W^{\prime }$	Attributes for policy update
$W^{u}$	Updated Policy
GID	Global Identifier
$S_{owner}$	Owner secret $t_{i}$ for setting access access control
U	Policy update parameter
IND−sCPA	Indistinguishability under selective CPA

**Table 3:** Users satisfying policy and access type (Partial/Full).

*U*	User Attribute Set *L*	L⊧W	$L⊧W^{u}$	DA
1	Doctor, Hospital-1, Clinic-X	Yes	Yes	F
2	Nurse, Hospital-2	No	No	P
3	Professor, University-1, University-2	Yes	Yes	F
4	Insurance-comp-1, Insurance-agent	Yes	No	F
5	Student, University-1	Yes	No	P

U: User, DA: Data Access, P: Partial, F: Full.

**Table 4:** Computational Costs Comparison with existing relevant schemes.

Scheme	Encryption	Decryption	Ciphertext Size
[5]	(5n+2)E	(2z+1)P+2zE	$|G_{T}\left|+\left(3n+1\right)|G\right|$
[6]	((K+2)n+3)E	3zP+zE	$|G_{T}\left|+\left(2n+1\right)|G\right|$
[7]	(3n+3)P+(5n+2)E	(3z+1)P+(z+1)E	$|G_{T}\left|+\left(3n+3\right)|G\right|$
[8]	(2n+2)E	(2z+1)P+2zE	$|G_{T}\left|+\left(2n+1\right)|G\right|$
[15]	$(2n+2p^{\prime })E$	(2z+1)P+zE	$\left(2n+p^{\prime }\right)\left|G\right|+p^{\prime }\left|G_{T}\right|$
[16]	$(3n+p^{\prime })E$	zP+zE	$n|G|+\left(n+p^{\prime }\right)\left|G_{T}\right|$
This Work	$(3p^{\prime })E$	2P	$2p^{\prime }\left|G\right|+p^{\prime }\left|G_{T}\right|$

*n*: number of attributes in access structure, *z*: users attributes satisfying policy, $p^{\prime }$: number of attribute sets in policy, *E*: Exponentiation, *P*: Pairing, *G*: Source group, i.e., g, $G_{T}$: Target group, i.e., *e*(*g*,*g*), *K*: depth of attribute vector.

**Table 5:** Comparison of schemes based on policy update.

Scheme	Encryption	Decryption	Ciphertext	Policy Update	Ciphertext Update
[26]	$3p^{\prime }E$	2P	$p^{\prime }\left(\left(u-n+1+t\right)|G|+\right|G_{T}\left|\right)$	$p^{\prime }tE$	(u−n−1)|G|
[27]	(3n+2)E	(2z+2)P+zE	$\left(2n+1\right)|G|+|G_{T}|$	$3nE+nZ_{p}$	$2n|G|+nZ_{p}$
[28]	(r+u+6)E	8E+6P	(6+u+r)|G|	(3t+1)E+P	(6+u+r)|G|
This Work	$3p^{\prime }E$	2P	$p^{\prime }\left(2|G|+\right|G_{T}\left|\right)$	$p^{\prime }2E$	$p^{\prime }\left(|G|+\right|G_{T}\left|\right)$

*E*: Exponentiation, *P*: Pairing, *G*: Source group, i.e., g, $G_{T}$: Target group, i.e., *e*(*g*,*g*), *r*: max number of revocated attributes, *u*: number of attributes in universe, *n*: number of attributes in access structure, *t*: number of revocated attributes from ciphertext.

**Table 6:** Shimmer Sensor Specification.

Source	Sampling Rate
Gyroscope	512 Hz
ECG	1024 Hz
EMG	512 Hz
GSR	128 Hz
Optical Pulse PPG	128 Hz

**Table 7:** Realtime Sensor’s Data Encryption.

Source	Encryption (ms)	Decryption (ms)	Ciphertext (bytes)
GSR	0.19	0.10	1813
Gyroscope	0.27	0.19	23,541
ECG	0.68	0.48	63,797
